# Supplementary material for: Reinstating plasticity and memory in a tauopathy mouse model with an acetyltransferase activator
Source: EMBO Mol Med. 2018 Oct 1;10(11):e8587. doi: 10.15252/emmm.201708587 (PMC6220301; doi:10.15252/emmm.201708587)

Table of contents

- **Appendix Supplementary Methods**
- **Appendix Supplemental reference**
- **Appendix Supplemental Figure S1 and S2 Legends**
- **Appendix Supplemental Figure S1 and S2**

Appendix Supplementary Methods

Drug production, testing and treatment.

Synthesis of carbon nanospheres of average size 400nm: 5 g of D(+) glucose (G8270-1KG, Sigma Aldrich) was dissolved in 50 mL of deionised water to form a clear solution, placed in a 65 mL teflon lined sealed stainless steel autoclave and maintained at 180C for 12 h. The products were isolated by centrifuging at 16000 rpm for 20 min. They were later purified by repeated washings using water and ethanol before being dried in the oven at 80C for 4 h. The final product is a brownish powder which had spheres with an average size of 400 nm (Supplemental Figure S2A).

Synthesis of TTK21: 2-Propoxy Benzoic Acid (3.3 mmol, CAS 2100-31-4, TCI Chemicals) was taken in DCM and treated with one eqivalent of thionyl chloride (3.66 mmol, 20920L05 S.D. Fine Chemicals Pvt. Ltd.) followed by the addition of 4-5 drops of DMF (Product # 12405) and the solution was refluxed for 4 h. Thionyl chloride was removed by evaporating the solvent 3-4 times and to that DCM was added (Solution A). 4-Chloro-3-(trifluoromethyl) amine (3.1 mmol) was dissolved in DCM and the solution was kept at 0C. To that triethylamine was added and was stirred for 30 min

(Solution B). The solution B was added dropwise to the solution A at 0C and the solution was heated to reflux for 4 h. The solution was evaporated to dryness and was purified by column chromatography. White crystalline solid was obtained.

Conjugation of TTK21 to CSP: To a suspension of CSP in DCM, one equivalent of $SOCl_2$ was added followed by the addition of few drops of DMF. The reaction mixture was refluxed at room temperature for 4 h. Thionyl chloride was removed by evaporating the solvent 3-4 times and to that DCM was added to make the suspension. One equivalent TTK21 was added to that solution followed by the addition of few drops of triethylamine. The reaction was refluxed for 4 h. The suspension was filtered and the precipitate was washed with DCM. The conjugated CSP-TTK21 was dried. The conjugation of TTK21 to the nanosphere was confirmed by Energy Dispersive X-ray Spectroscopy EDX (Supplemental Figure S2B). The spectrum of CSP-TTK21 shows that wt % of Fluorine is 0.35 which belongs to TTK21 indicating the covalent attachment of TTK21 to CSP.

Western blot analysis: using an antibody against acetylated-H3K9 (raised in-house; 1/2000; Modak et al, 2012). Loading control was checked using H3 histone antibody (raised in-house; 1/3000; Modak et al, 2012). The expression levels were quantified by Image Analyzer software (Supplemental Figure S2C).

Immunofluorescence: C57BL/6 male mice were intraperitoneally injected with a suspension of 2mg/ml of CSP or CSP-TT21 (500 µg each). After 72 h the mice were sacrificed by cervical dislocation. Their brains were rapidly removed, brain tissue was fixed with paraformaldehyde and cryoprotected in a 20% sucrose solution overnight before storage at −20C. Coronal sections were made through the dorsal hippocampus using a Vibratome (VT1000M; Leica). The tissue sections (20 µm) were permeabilized in 1× PBS/Triton X-100 2% for 30 min. Nonspecific labeling was

blocked by 1× PBS/0.1% Triton X-100/5% horse serum for 45 min at room temperature. The tissues were immunolabelled with H3K14ac (1/250 diluted in 0.1% Triton-X/PBS) overnight at 4C. The sections were washed with 1× PBS for 3 times followed by the incubation with the anti-rabbit Alexa-Flour 568 secondary antibody for 1 h at room temperature. After 3 washes with 1× PBS, the nuclei of the sections were stained with hoechst (1/1000 dilution) for 5 min. After two PBS washes, the sections were mounted. The images were acquired with a Zeiss LSM880 confocal imaging system (Supplemental Figure S2D).

Drug treatments

CSP and CSP-TTK21 molecules were stored at -20C in multiple aliquots. CSP or CSP-TTK21 was resuspended in injectable water at a final concentration of 2mg/ml and sonicated for 6-8 s. Animals were randomized on body weight and injected intraperitoneally (*i.p*) with the molecules at the dose of 20mg/kg of body weight.

Stereotaxic lentivirus-GFP injections for GFP staing of dendritic spines.

The pRRLSIN.cPPT.PGK-GFP.WPRE originated from the Trono Lab and was obtained through Addgene (plasmid #12252). For stereotaxic lentivirus-GFP injections, mice that had been maintained in home cage (basal conditions) were anesthetized with 4-5% Isoflurane at flow rate of 2L/min (1L/min of air and 1L/min of oxygen) for 1-2 minutes until they completely lost consciousness. Their head was fixed on the stereotaxic apparatus and a mask connected to maintained anesthetic with 2.0-2.5% Isoflurane during the entire surgery. Mice were injected with 150 nl of lentivirus-GFP PGK solution, delivered at 0.1 ml/min, into the CA1 according to stereotaxic coordinates (Krezymon et al, 2013) Bregma: antero-posterior -2.0 mm, lateral +1.5 mm, dorso-ventral -1.5 mm. Incised skin was sutured with sterile stitching

fibers. After surgery, mice were placed in a heated chamber to recover before returning to their home cage.

Primer sequences used in RT-qPCR were as follows:

Kl-F: GTTGCCCACAACCTACTTTTG
Kl-R: GGAGCTTAAGGCGATAGACAC

Nts-F : GTGTGCCTGACTCTCCTG
Nts-R : GCAAGGTCATTTTCCAAGACG

Mvd-F : TCAGTCATGGCCTCAGAAAAG
Mvd-R : TCTGTGAAGTCCTTGCTAATGG

Nr4a1-F: AAA-ATC-CCT-GGC-TTC-ATT-GAG
Nr4a1-R: TTT-AGA-TCG-GTA-TGC-CAG-GCG

Egr1-F : TAT-GAG-CAC-CTG-ACC-ACA-GG
Egr1-R : GAG-TCG-TTT-GGC-TGG-GAT-AAC

Arc-F : AGC-AGC-AGA-CCT-GAC-ATC-CT
Arc-R : GTG-ATG-CCC-TTT-CCA-GAC-AT

Cyp46a1-F : GGCTAAGAAGTATGGTCCTGTTGTAAGA
Cyp46a1-R : GGTGGACATCAGGAACTTCTTGACT

Kcnh3-F : CGGATCCAAAGGCTTCAATG
Kcnh3-R: AGCAGGATAAAGGGTGACTTC

Itgax-F: AAG-CAG-AGC-CAG-AAC-TTC-CC
Itgax-R: AAG-GGC-TTG-ACG-TGG-AGA-TG

Ccl4-F: GCC-CTC-TCT-CTC-TCC-TCT-TGC-T
Ccl4-R: GAG-GGT-CAG-AGC-CCA-TTG

Gfap-F : CGC-GAA-CAG-GAA-GAG-CGC-CA
Gfap-R: GTG-GCG-GGC-CAT-CTC-CTC-CT

PolII-F: AAT-CCG-CAT-CAT-GAA-CAG-TG
PolII-R: TCA-TCC-ATT-TTA-TCC-ACC-ACC

hTau-F: GTA-CGG-GTT-GGG-GGA-CAG-GA
hTau-R: CCC-GGT-TCC-TCA-GAT-CCG-TC

Dusp1-F : GGA-GGA-TAT-GAA-GCG-TTT-TCG-G
Dusp1-R : GGA-TTC-TGC-ACT-GTC-AGG-CAC-A

cFos-F : CGG-GTT-TCA-ACG-CCG-ACT-A
cFos-R : TTG-GCA-CTA-GAG-ACG-GAC-AGA

36b4-F : ACT-GGT-CTA-GGA-CCC-GAG-AAG
36b4-R : TCC-CAC-CTT-GTC-TCC-AGT-CT

Appendix Supplemental reference

Krezymon A, Richetin K, Halley H, Roybon L, Lassalle JM, Frances B, Verret L, Rampon C (2013) Modifications of hippocampal circuits and early disruption of adult neurogenesis in the tg2576 mouse model of Alzheimer's disease. *PloS one* 8: e76497

Modak R, Das Mitra S, Vasudevan M, Krishnamoorthy P, Kumar M, Bhat AV, Bhuvana M, Ghosh SK, Shome BR, Kundu TK (2014) Epigenetic response in mice mastitis: Role of histone H3 acetylation and microRNA(s) in the regulation of host inflammatory gene expression during Staphylococcus aureus infection. *Clinical epigenetics* 6: 12

Appendix Supplemental Figure S1 Legend

RT-qPCR validations of RNA-seq data.

A RNA-seq data result from the cohort of mice described in Fig 3,4. Statistical analysis was performed with the method proposed by Love et al. (2014) (Love et al, 2014) implemented in the DESeq2 Bioconductor library (v1.0.19). ***Mvd***: $ pathology p=1,17E-27 for WT VEH vs. TAU VEH; ***Cyp46a1***: $ pathology p=1,80E-13 for WT VEH vs. TAU VEH, * learning p=0,0168 for WT VEH vs. WT VEH_HC, # molecule p=4.00E-05 for TAU MOL *vs.* TAU VEH; ***Kcnh3***: $ pathology p=1.06E-27 for WT VEH vs. TAU VEH, # molecule p=0.0054 for TAU MOL *vs.* TAU VEH; ***cfos***: $ pathology p=0.0276 for WT VEH vs. TAU VEH, * learning p=2.5E-27 for WT VEH *vs.* WT VEH_HC, # molecule p=0.0016 for TAU MOL *vs.* TAU VEH; ***Egr-1***:

$ pathology p=1.66E-06 for WT VEH vs. TAU VEH, * learning p=8.69E-12 for WT VEH *vs.* WT VEH_HC, # molecule p=1.19E-06 for TAU MOL *vs.* TAU VEH; ***Klotho, Kl***: * learning p=2.78E-22 for WT VEH *vs.* WT VEH_HC, $ pathology p=1.73E-32 for WT VEH *vs.* TAU VEH, # molecule p=1.73E-14 for TAU MOL *vs.* TAU VEH; ***Nts***: $ pathology p=0.0110 for WT VEH *vs.* TAU VEH, # molecule p=7.34E-06 for TAU MOL *vs.* TAU VEH.

B RT-qPCR result from another cohort of mice having received the same treatment as in A (n=5/group). One-way ANOVA with uncorrected Fisher's test. ***Mvd***: F(3,16)=31.69, p<0.0001; $ pathology p=0.0001 for WT VEH vs. TAU VEH; ***Cyp46a1***: F(3,14)=1.777, p=0.1977; ***Kcnh3***: F(3,16)=2.469, p=0.0993; $ pathology p=0.0222 for WT VEH vs. TAU VEH, (*) learning indicates a tendency p=0.0695 for WT VEH *vs.* WT VEH_HC; ***cfos***: F(3,16)=12.08, p=0.0002; * learning p=0.0002 for WT VEH *vs.* WT VEH_HC; ***Egr-1***: F(3,16)=15.99, p<0.0001; * learning p=0.0001 for WT VEH *vs.* WT VEH_HC. ***Klotho, Kl***: F(3,16)=2,949, p=0.0643; * learning p=0.0145 for WT VEH *vs.* WT VEH_HC, $ pathology p=0.0395 for WT VEH *vs.* TAU VEH. ***Nts***: F(3,14)=4,290, p=0.0241; $ pathology p=0.0036 for WT VEH *vs.* TAU VEH, # molecule p=0.0081 for TAU MOL *vs.* TAU VEH.

C RNA-seq data result from the cohort of mice described in Fig 1C. Statistical analysis was performed with the method proposed by Love et al. (2014) (Love et al, 2014) implemented in the DESeq2 Bioconductor library (v1.0.19). * when WT VEH is significantly different from TAU VEH. ***Arc***: p=8,12E-15; ***c-Fos***: p=1,95E-08; ***Egr-1***: p=2,57E-12; ***Dusp1***: p=8,35E-09; ***Nr4a1***: p=5,26E-56 .

D Time line of the experiment with chronic injections in THY-Tau22 mice: 8 month-old mice were injected 10 times (1 per 2 weeks) with Vehicle (WT mice, WT VEH, saline), Vehicle (THY-Tau22 mice (TAU VEH), CSP 500 µg /mouse) or Molecule

(THY-Tau22 mice (TAU MOL), CSP-TTK21 500 µg/mice), starting at the age of 3 months. Mice were tested for spatial memory in the Morris water maze 5 days after the last injection, probe test was performed 10 days after and mice were killed 48hr after. RNA extracts were isolated from the dorsal hippocampus (n=6/group).

E RT-qPCR data resulting from the cohort of mice in B (n=6/group). One-way ANOVA with uncorrected Fisher's test. ***Arc***: F(2,15)=12.55, p=0,0006 ; $ pathology p=0.0002 for WT VEH vs. TAU VEH, # molecule p=0.0047 for TAU MOL *vs.* TAU VEH; ***c-Fos***: F(2,15)=1.323, p=0.2957, ns; ***Egr-1***: F(2,15)=4.349, p=0.0324; $ pathology p=0.0487 for WT VEH vs. TAU VEH, # molecule p=0.0128 for TAU MOL *vs.* TAU VEH; ***Dusp1***: F(2,15)=0.2095, p=0.8133; ns; ***Nr4a1***: F(2,15)=3.925, p=0.0426; $ pathology p=0.0297 for WT VEH vs. TAU VEH, # molecule p=0.0270 for TAU MOL *vs.* TAU VEH.

Appendix Supplemental Figure S2 Legend

Drug production and testing.

A Field emission scanning electron microscopy (FESEM) images of CSP molecules showing carbon nanospheres of average size 400nm. Scale bar: 3 µm.

B Energy Dispersive X-ray analysis of CSP-TTK21 shows the presence of fluorine, thereby confirming the conjugation of TTK21 to CSP.

C Western Blot analysis was performed on lysates obtained from U87 cells treated for 12h with growing concentrations of CSP and CSP-TTK21 (50, 100, 150 µg/ml). CSP-TTK21 induced a significant increase in H3K9ac compared to CSP-treated cells (n= 3; p = 0.03; student's t test). A representative image is shown as a juxtaposition of images obtained on a different part of the gel (see Source Data Appendix).

D CSP-TTK21 induces histone acetylation in the dorsal hippocampus. Immunofluorescent labeling of 20 µm brain sections using H3K14ac antibody perfomed in WT mice upon treatment with CSP or CSP-TTK21 for 3 days. Scale bar: 200 µm.

Appendix Supplemental Figure S1

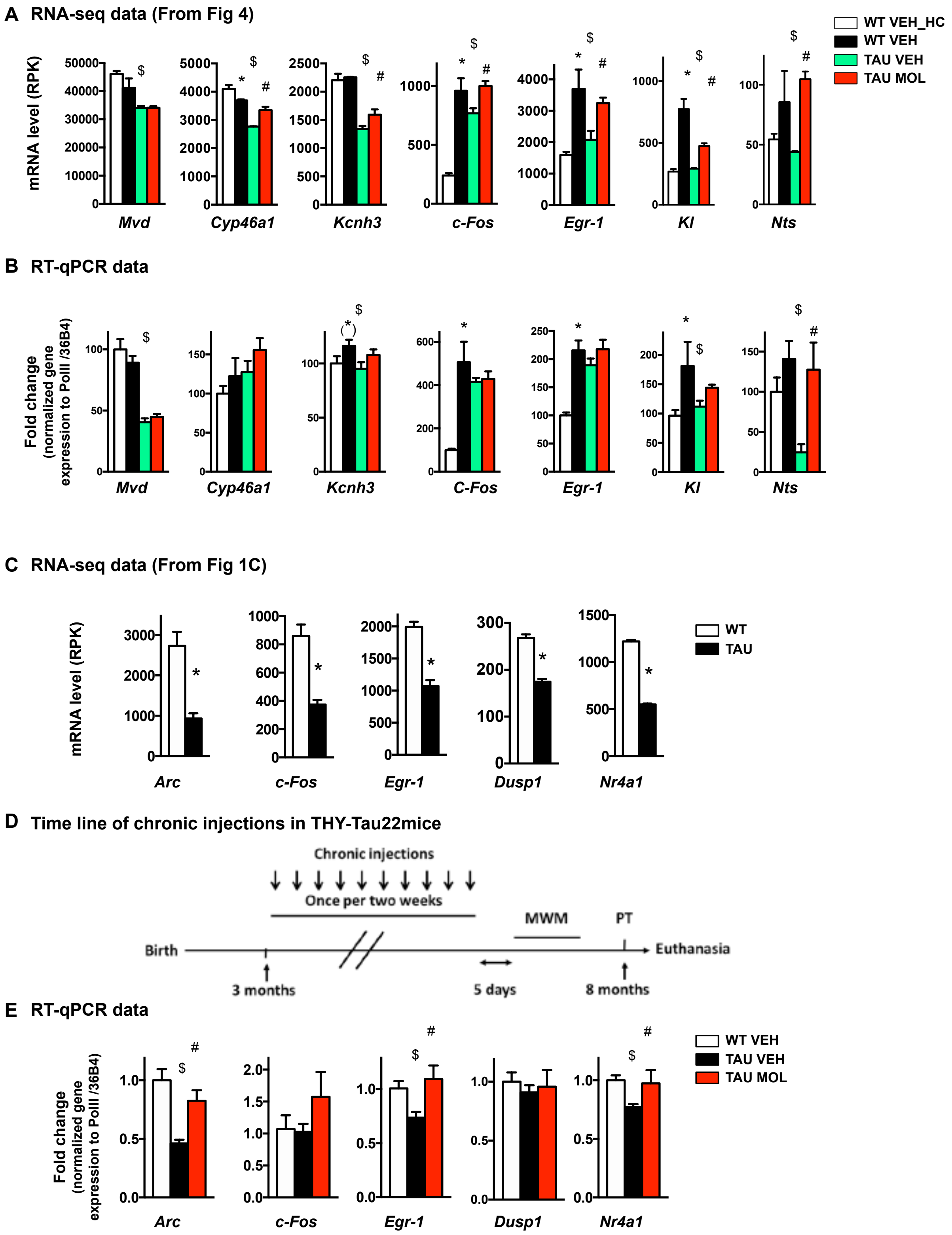

Appendix Supplemental Figure S2

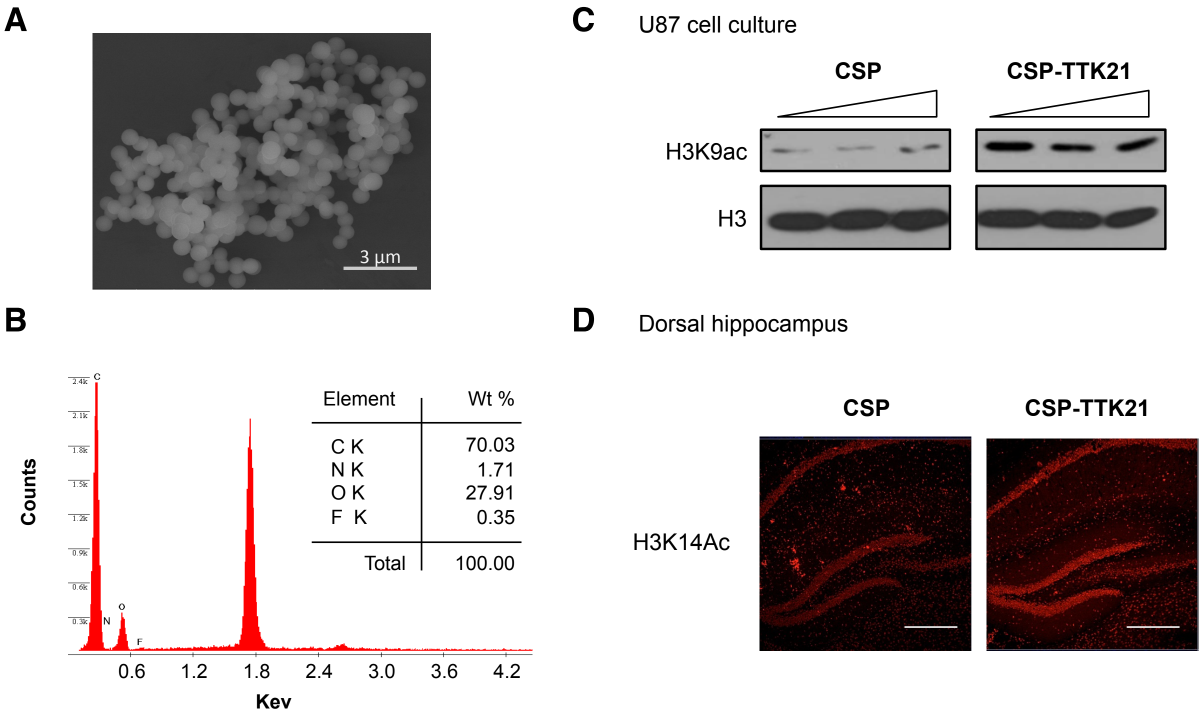

Supplement: Supplementary file 1 — Appendix [file EMMM-10-e8587-s001.pdf]
