# Supplementary material for: Reinstating plasticity and memory in a tauopathy mouse model with an acetyltransferase activator
Source: EMBO Mol Med. 2018 Oct 1;10(11):e8587. doi: 10.15252/emmm.201708587 (PMC6220301; doi:10.15252/emmm.201708587)
Supplement: Supplementary file 2 — Expanded View Figures PDF [file EMMM-10-e8587-s002.pdf]

Expanded View Figures

Figure EV1. CSP-TTK21 treatment of THY-Tau22 mice improves long-term spatial memory retention and induces some histone acetylation in the hippocampus.

A Western blot analyses of dorsal hippocampal extracts from 12-month-old THY-Tau22 vs. aged-matched WT controls. Levels of different histone modifications were tested. Only the tetra-acetylated form of H2B (K5K10K15K20) shows a significant decrease in tauopathic mice. Normalization was performed on the total amount of proteins transferred onto the membranes, and data are presented as fold induction of the acetylated histone/total H2B histone ratio. n = 5–6/group as noted. Multiple t-tests, and H2BK5K10K15K20, *P = 0.0047 for THY-Tau22 vs. WT mice.

B Scatter plot presenting RNA-seq data comparison between THY-Tau22 vs. WT mice. The $\log_2$(Fold-Change) was estimated by DESeq2. Red dots correspond to genes with adjusted P-value < 0.05.

C Habituation consisted of one trial to the visible platform. No statistical difference was observed in the distance from the habituation platform depending on the Group (F(1,37) = 0.17, P = 0.84).

D Distance to reach the platform (in meters) during the acquisition period. All groups of mice displayed significant and similar decreased of the distance traveled to reach the platform depending on the day [Day effect, F(4,148) = 26.47, P < 0.001; Group and Group × Day effects, ns]. Two-way ANOVA followed by a multiple-comparisons test (Newman–Keuls).

E The swim speed during the acquisition was the same for all groups [Day effect, (F(4,148) = 0.95, P = 0.46) and Group effect (F(2.37) = 1.06, P = 0.35)]. Two-way ANOVA followed by a multiple-comparisons test (Newman–Keuls).

F The number of visit to the previous platform location showed a significant difference in the number of crossing performed into it, and the three others for WT VEH mice (t(17) = 2.62, P < 0.05) and TAU MOL (t(13) = 2.70, P < 0.05) while there was no difference for the TAU VEH (t(10) = 0.21, P = 0.83). One-way ANOVA, Student's t-test, # P < 0.05 vs. mean of three others.

G The latency to first visit to the target platform compared to the mean latencies to the first visit to three others platform locations during the probe trial highlighted a global difference [Group Effect (F(2.37) = 5.87, P = 0.008) due to TAU VEH mice that significantly differed from the two others groups (TAU VEH vs. WT VEH (P = 0.004) and vs. TAU MOL (P = 0.012)]. There was a significant difference between the latencies to the first visit to the target platform with the three others for WT mice (t(17) = 6.90, P < 0.001) and TAU MOL (t(13) = 2.30, P < 0.05) while there was no difference for the TAU MOL (t(10) = 0.82, P = 0.42). One-way ANOVA, *post hoc* analyses *P < 0.05 vs. TAU VEH and Student's t-test, #P < 0.05 vs. mean of three others.

H Tracks representing the platform search during retention is shown for one mouse from each group that was either closest to the mean track or best of the group. The result of the probe test is noted below (in seconds).

I Western blot analyses were performed 22 days post-injection from experiment shown in Fig 1D, on n = 5 mice/group. Levels of the different histone modifications were tested. Normalization was performed on the total amount of proteins transferred onto the membranes and data are presented as fold induction of the acetylated histone/total H2B histone ratio. Multiple t-tests, and H2BK5K10K15K20ac, *P = 0.0006 and H3K27ac, *P = 0.0054 for CSP vs. CSP-TTK21, (#) indicates a tendency (P = 0.0736).

Data information: Graphs are mean ± SEM. (C–G) Analyses of different parameters on the mice used in the Morris water maze experiment described in Fig 1 (WT VEH, n = 17, TAU VEH, n = 10 and TAU MOL, n = 13).

© 2018 The Authors

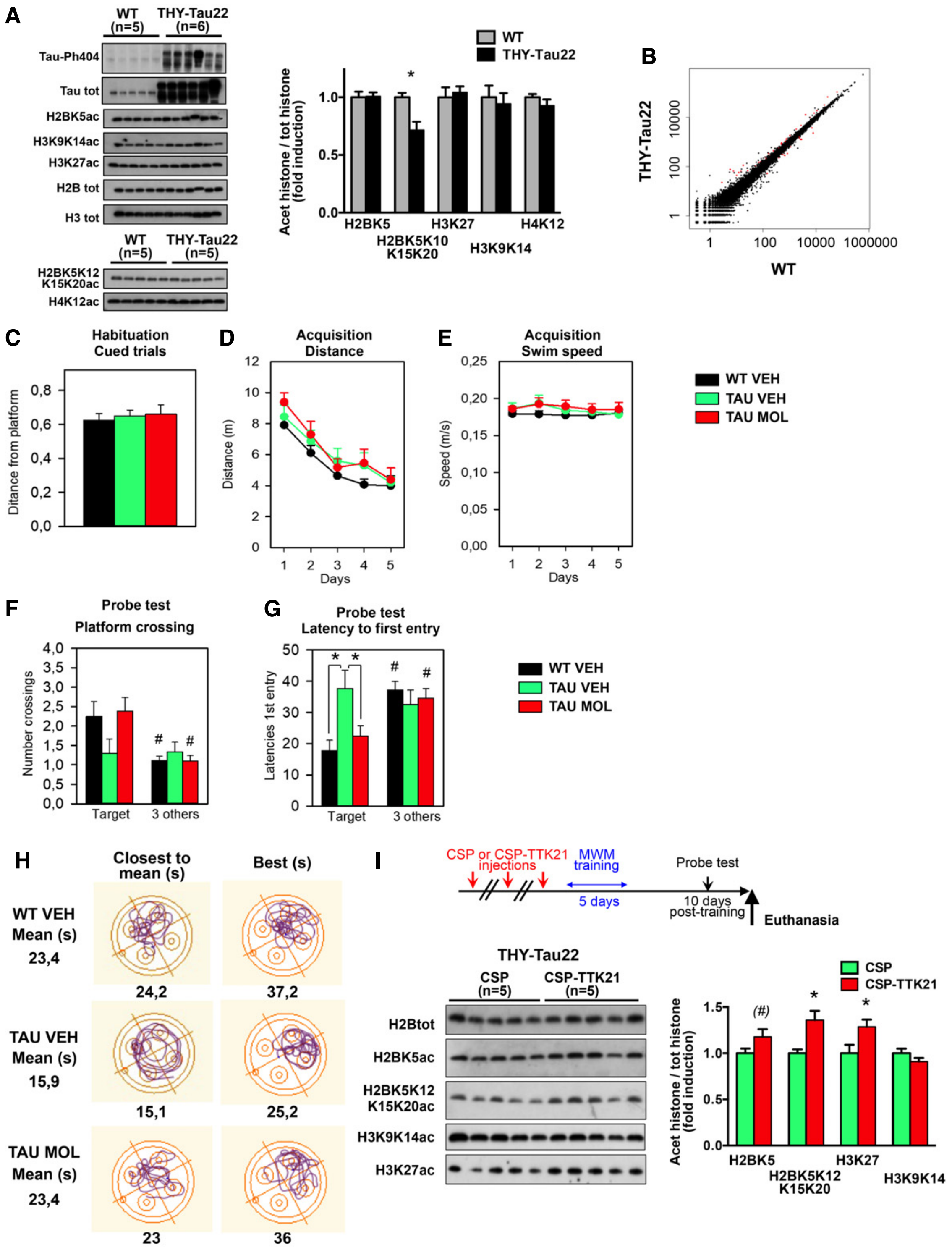

Figure EV1.

© 2018 The Authors

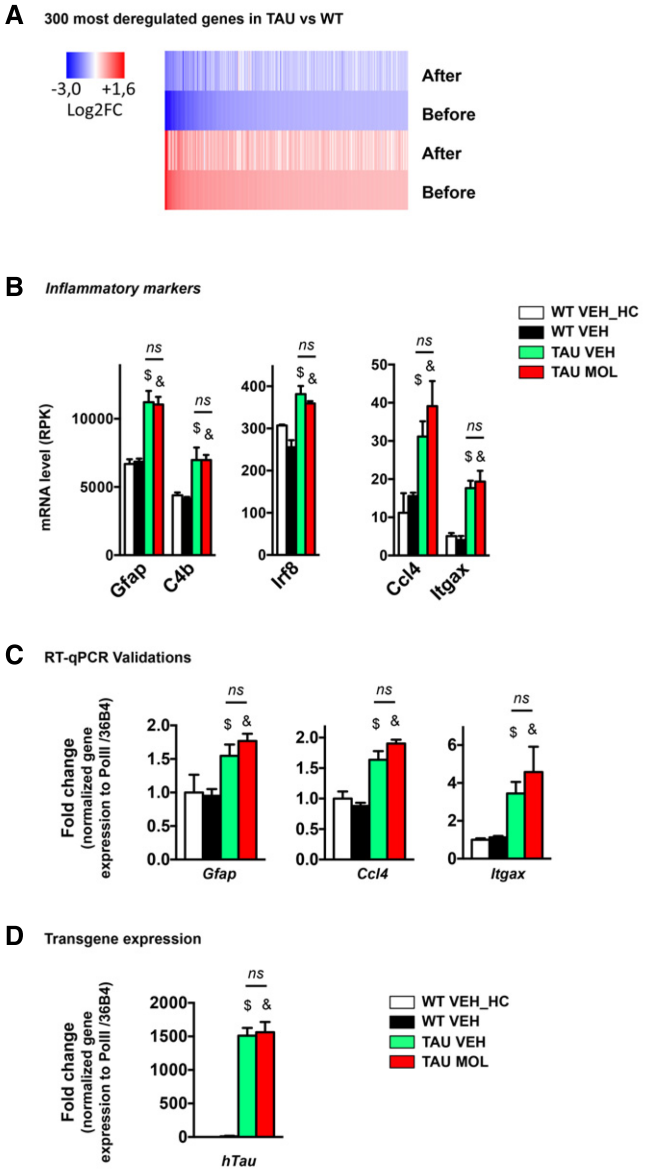

Figure EV2. CSP-TTK21 normalizes most deregulated genes in TAU mice but does not affect inflammatory processes, nor the human Tau transgene expression.

A Fold changes of the 300 most deregulated genes in TAU vs. WT mice from the RNA-seq study (adjusted *P*-value < 0.05) represented as a heatmap before and after the treatment. Color coding was performed according to the $\log_2$(Fold-Change) for down (blue)- and up (red)-regulated genes. Fold changes tend to go back to normal values (zero) after the treatment. Comparisons: before treatment, TAU VEH vs. WT VEH; after treatment, TAU MOL vs. WT VEH.

B RNA-seq data of inflammatory markers. RNA-seq data (n = 2–3/group) showing that expression of a series the inflammation-related genes significantly upregulated in tauopathic vs. WT mice is not changed upon CSP-TTK21 treatment of THY-Tau22 mice. Adjusted *P*-value < 0.05, $ TAU VEH vs. WT VEH; & TAU MOL vs. WT VEH mice; $ TAU VEH vs. WT VEH_HC, & TAU MOL vs. WT VEH_HC, ns non-significant when TAU MOL is compared to TAU VEH. See Materials and Methods for statistical analyses of RNA-seq data (n = 2–3/group).

C RT–qPCR validations performed in a different cohort of mouse from the RNA-seq study (n = 4–5/group). One-way ANOVA with uncorrected Fisher's test. *Gfap*: $F(3,14) = 6.615$, $P = 0.0052$. $ $P = 0.0207$ for WT VEH vs. TAU VEH, & $P = 0.0020$ for TAU MOL vs. WT VEH, ns non-significant when TAU MOL is compared to TAU VEH, $P = 0.3470$. *Ccl4*: $F(3,14) = 22.35$, $P < 0.0001$, $ $P = 0.0001$ for WT VEH vs. TAU VEH, & $P = 0.0001$ for TAU MOL vs. WT VEH, ns non-significant when TAU MOL is compared to TAU VEH, $P = 0.0915$. *Itgax*: $F(3,15) = 5.127$, $P = 0.0122$. $ $P = 0.0472$ for WT VEH vs. TAU VEH, & $P = 0.0058$ for TAU MOL vs. WT VEH, ns non-significant when TAU MOL is compared to TAU VEH, $P = 0.3078$.

D CSP-TTK21 does not influence the Tau transgene expression as evaluated by RT–qPCR in the different experimental groups (n = 5/group). One-way ANOVA with uncorrected Fisher's test: *hTau* $F(3,15) = 75.61$, $P < 0.0001$. $ $P = 0.0001$ for WT VEH vs. TAU VEH, & $P = 0.0001$ for TAU MOL vs. WT VEH, ns non-significant when TAU MOL is compared to TAU VEH, $P = 0.7240$.

© 2018 The Authors

Figure EV3. H2Bac ChIP-seq replicate comparisons.

A Three groups of mice were injected three times (1 per week) with NaCl (WT VEH), CSP (vehicle, VEH) or CSP-TTK21 (molecule, MOL) (500 µg/mice; THY-Tau22 mice (TAU) before euthanasia. ChIP-seq analyses performed with H2Bac (replicates: rep1, rep2) and H3K27ac (rep2) on the dorsal hippocampus of WT VEH, TAU VEH, and TAU MOL mice. The table shows the number of aligned reads per samples obtained after sequencing. Inputs were sequenced in each replicate.

B Principal component analyses (PCA). For each condition (WT VEH, TAU VEH, TAU MOL), islands detected with SICER in both replicates were intersected using BEDTools (Quinlan & Hall, 2010) intersect v26. Then, all intersected regions, one dataset per condition, were combined using BEDTools merge v26 to obtain a dataset containing all regions bound in at least one condition. The number of reads falling into the union dataset was computed using BEDTools intersect v26. Data were normalized using the method proposed by Anders and Huber (2010). For PCA plotting, data were transformed in order to stabilize variance using the DESeq2 28/04/y 18:23Bioconductor package (Love *et al*, 2014).

C Mean H2Bac profiles established with SeqMiner for all genes at TSS (top) and gene bodies (bottom) for the first quartile (poorly expressed genes) in the two replicates (rep1 and rep2). Significance (Benjamini, $P < 0.05$) of the annotations associated with both TSS (green) or gene profiles (red) is shown (DAVID, KEGG pathways).

D Mean H2Bac profiles established separately for each replicates (rep1 and rep2) with SeqMiner for the 1,624 decreased peaks obtained in the comparison TAU VEH vs. WT VEH (left) and TAU MOL vs. WT VEH (right).

E Venn diagrams showing differentially regulated peaks separately, either the decreased peaks (Left) or the increased peaks (right), before (TAU VEH vs. WT VEH) and after (TAU MOL vs. TAU VEH) the treatment, for all peaks with an FDR < 0.001.

© 2018 The Authors

A

Number of reads per samples

Sample name	Aligned reads rep1	Aligned reads rep2
Input WT	31 177 645	46 880 211
Input Tau CSP	29 301 904	44 089 030
Input Tau CSP-TTK21	29 518 993	34 591 548
H2Bac WT	38 741 338	49 583 351
H2Bac Tau CSP	34 314 567	49 481 027
H2Bac Tau CSP-TTK21	32 620 702	45 241 712
H3H27ac WT HD	/	48 009 848
H3H27ac Tau CSP	/	41 871 775
H3H27ac Tau CSP-TTK21	/	39 898 984

B

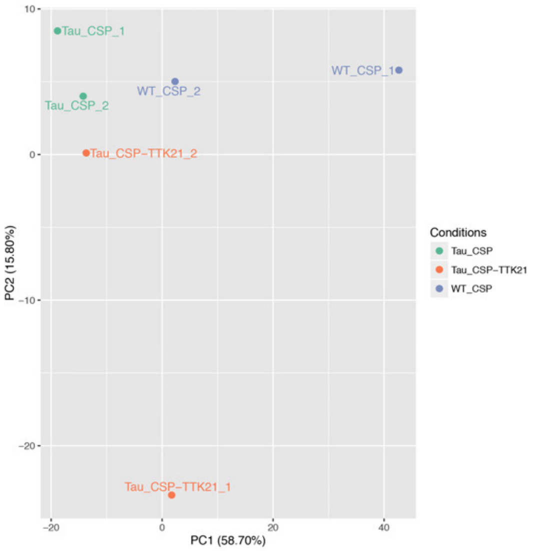

C

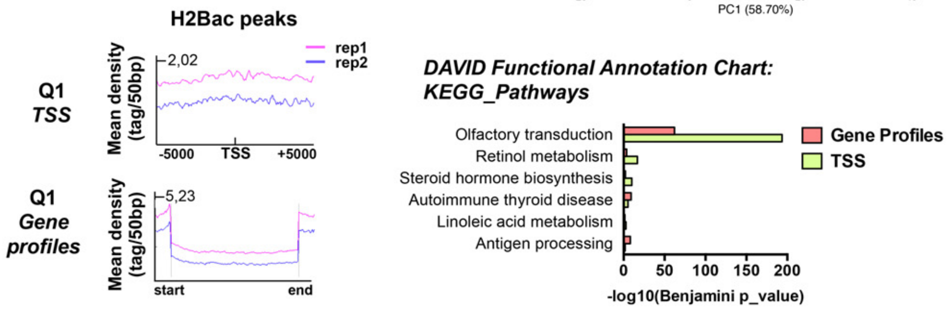

D

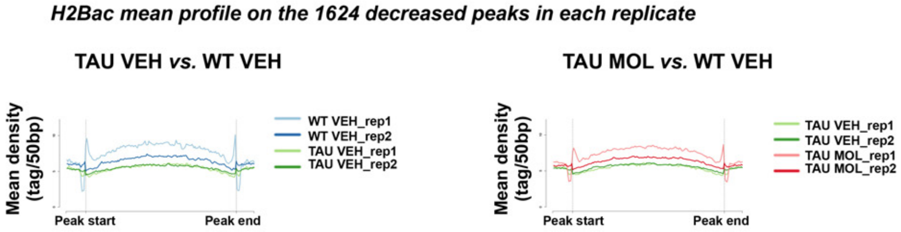

E

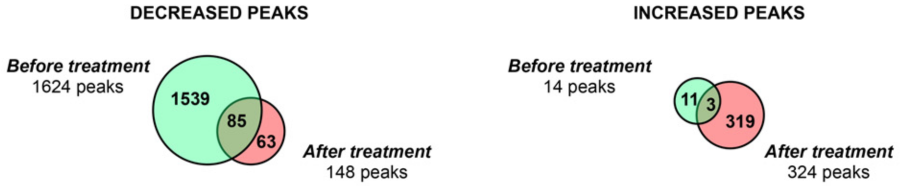

Figure EV3.

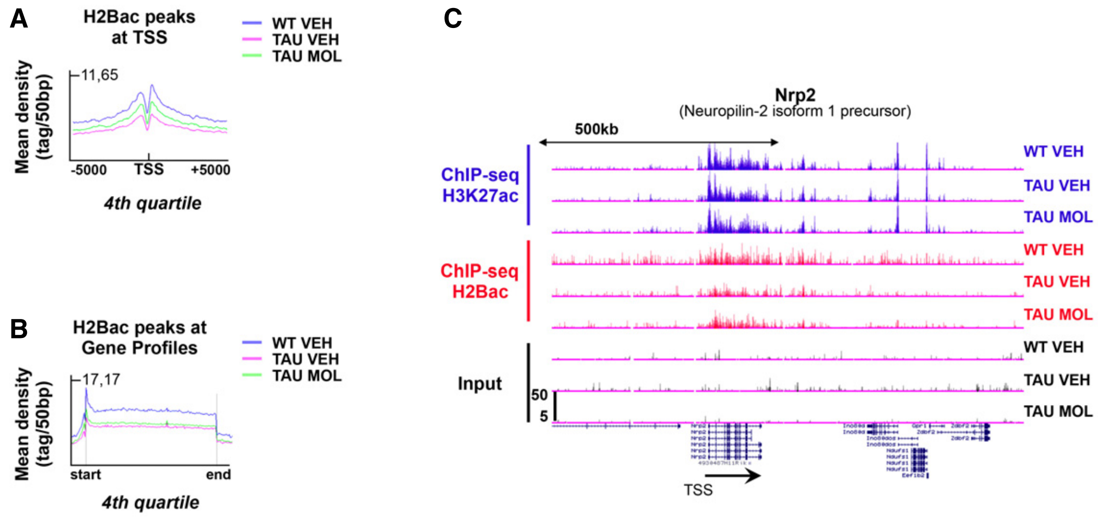

Figure EV4. Differential analyses of H2Bac enrichment at the TSS and gene profiles of the fourth-quartile genes.

A Mean profiles established with SeqMiner for the mostly expressed genes (fourth quartile) at TSS for H2Bac.

B Mean H2Bac profiles established with SeqMiner for the mostly expressed genes (fourth quartile) at the gene profiles. The severe decrease in H2Bac enrichment is poorly compensated at the gene profiles, only a few genes showing significant recovery, such as the *neuropilin-2 isoform 1 precursor* (*Nrp2)* gene locus. Nrp2 is a receptor for semaphorin 3F, which together act as a strong axonal repellent and pruning factor for hippocampal axons in the developing nervous system; their role in the postnatal brain is thought to direct hippocampal neural circuit formation (Shiflett *et al*, 2015).

C UCSC genome browser view showing H3K27ac ChIP-seq (blue), H2Bac ChIP-seq (red), and input (black) signals around the *Nrp2* gene locus. H3K27ac displays a broad profile along the gene body. H2Bac presents a similar broad profile on this gene. Differential peak calling analyses revealed that the H2Bac signature is downregulated at this locus in tauopathic mice compared to WT mice (intron, FDR = 0.0026) and upregulated by CSP-TTK21 treatment of THY-Tau22 mice (intron, FDR = 3.8×10^{-07}; intron, FDR = 4.8×10^{-09}; distant promoter, FDR = 9.65×10^{-09}).

 © 2018 The Authors
