# Supplementary figures and images for: Reinstating plasticity and memory in a tauopathy mouse model with an acetyltransferase activator
Source: EMBO Mol Med. 2018 Oct 1;10(11):e8587. doi: 10.15252/emmm.201708587 (PMC6220301; doi:10.15252/emmm.201708587)

Source data for Appendix  
(Appendix Supplemental Figure S2)

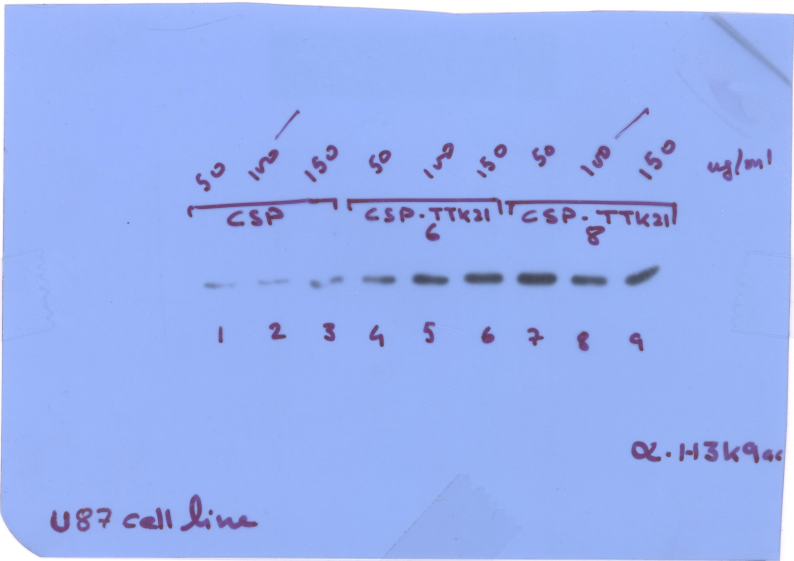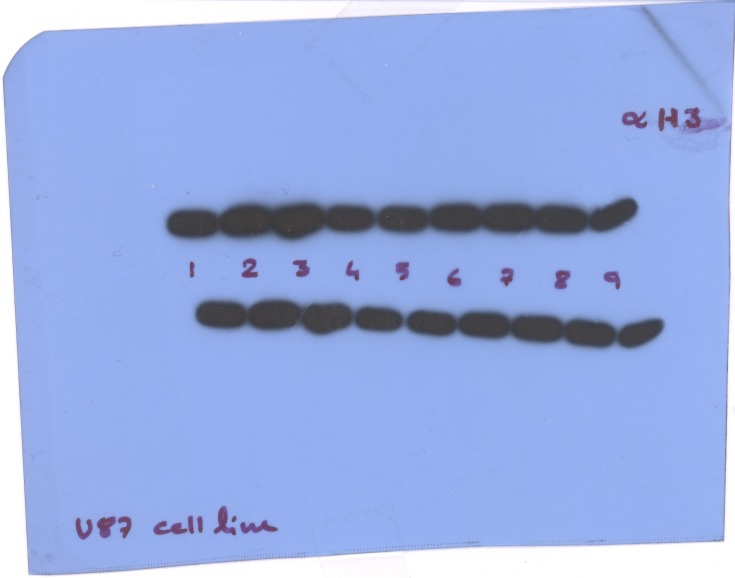

Supplement: Supplementary file 3 — Source Data for Appendix [file EMMM-10-e8587-s004.pdf]
