# Supplementary material for: Reinstating plasticity and memory in a tauopathy mouse model with an acetyltransferase activator
Source: EMBO Mol Med. 2018 Oct 1;10(11):e8587. doi: 10.15252/emmm.201708587 (PMC6220301; doi:10.15252/emmm.201708587)

Source data for Klotho
(Figure 4G)

Image Lab™ Software

• Stain-free gel

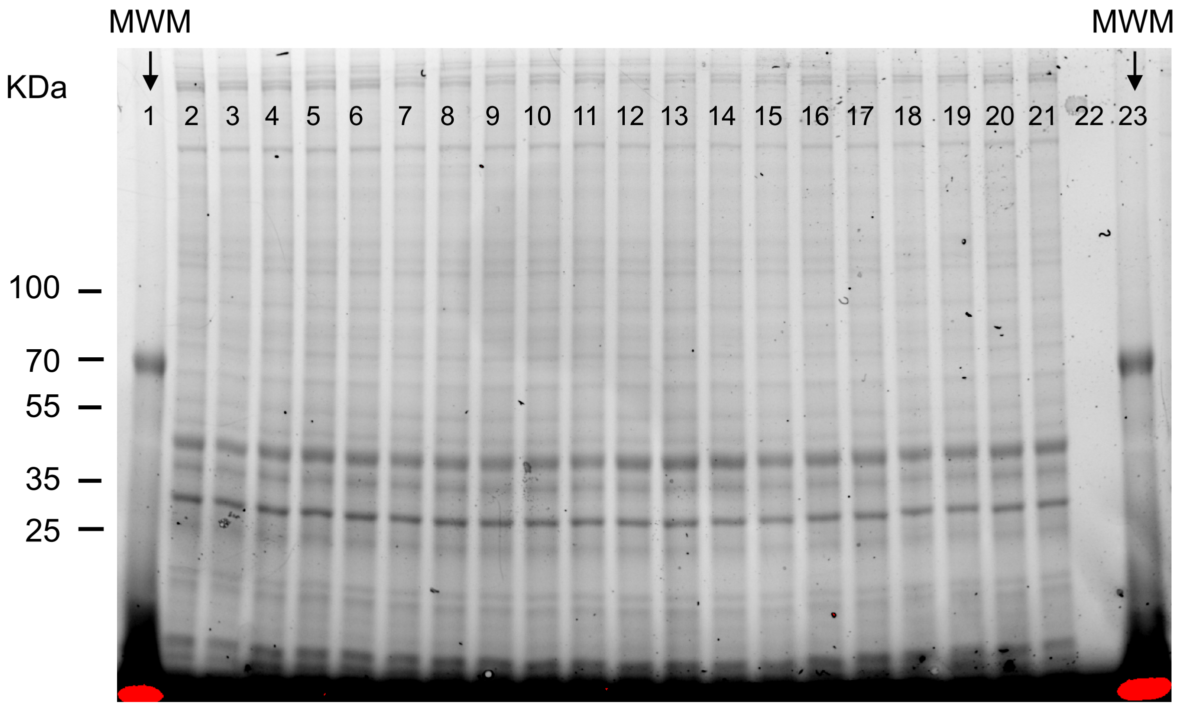

• Western blot: **Klotho** (Ref#AF1819; R&D Systems)

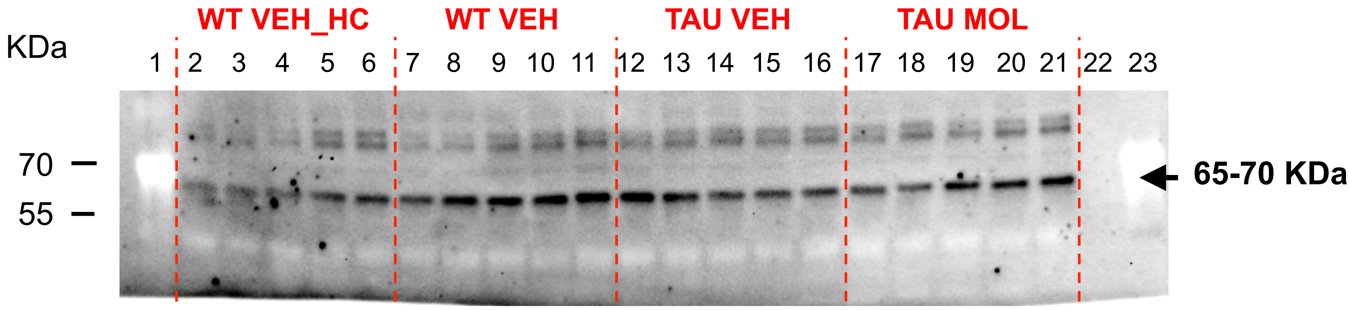

Source data for Neurotensin
(Figure 4G)

Image Lab™ Software

• Stain-free nitrocellulose

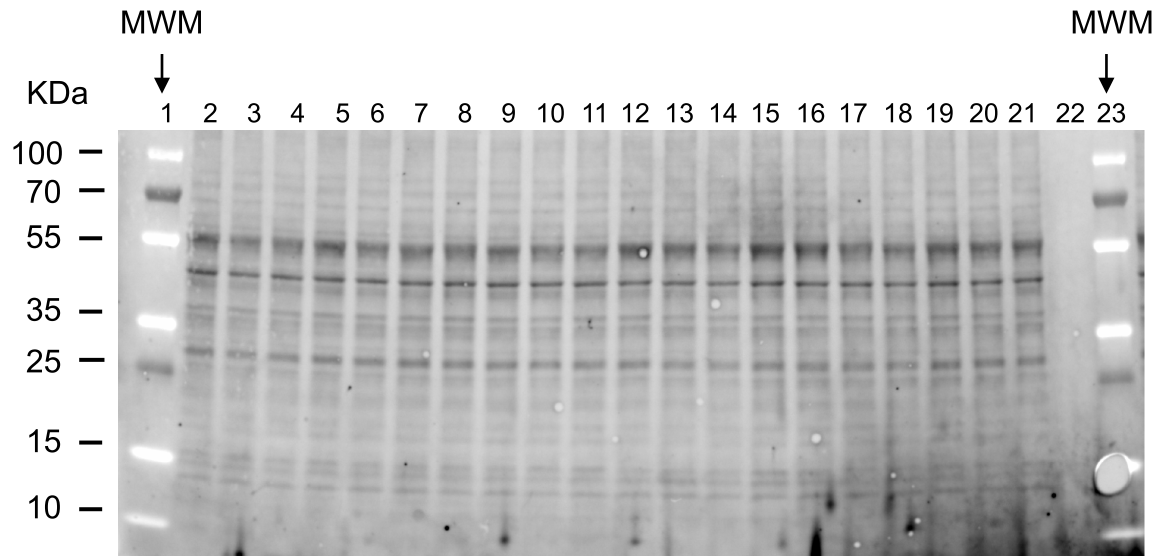

• Western blot: **Neurotensin** (Ref#BML-NA1230; Enzo Life Sciences)

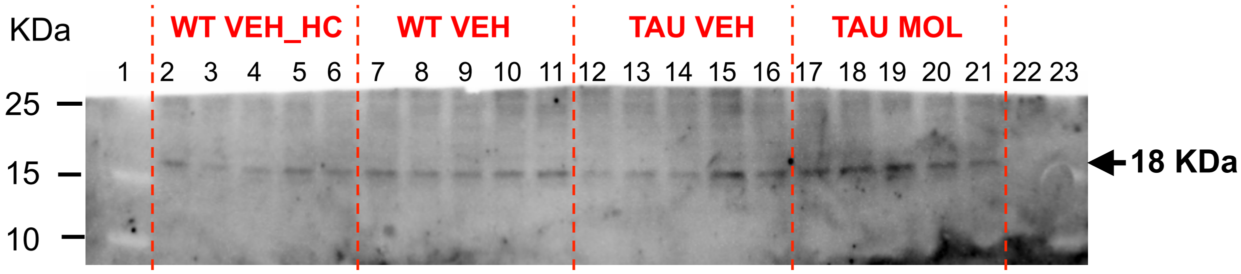

Source data for Actin
(Figure 4G)

Image Lab™ Software

• Stain-free nitrocellulose

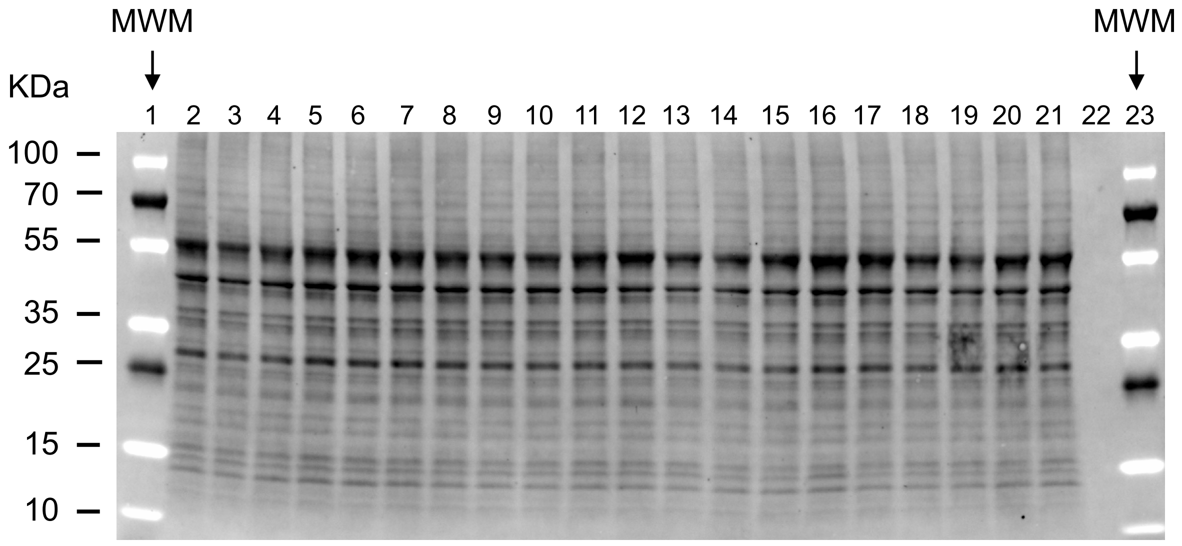

• Western blot: **Actin** (Ref#A2066; Sigma)

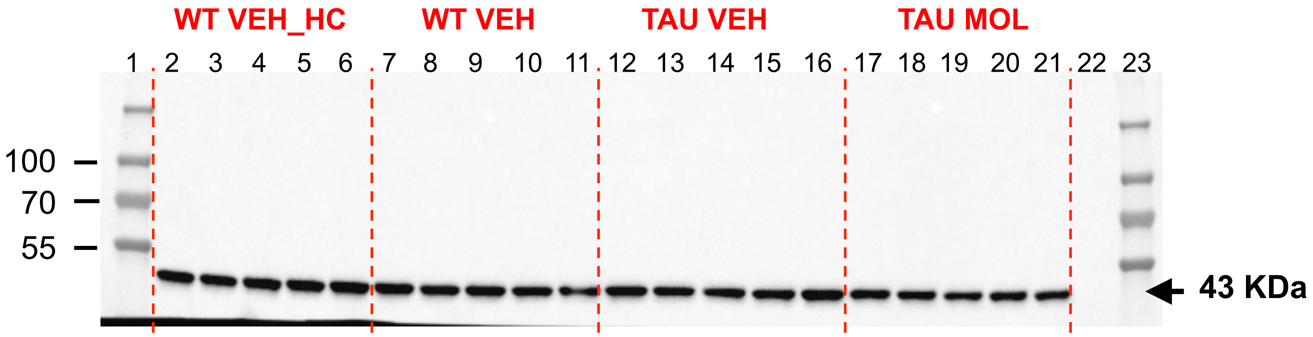

Supplement: Supplementary file 5 — Source Data for Figure 4 [file EMMM-10-e8587-s003.pdf]
